# Micronutrient-Fortified Milk and Academic Performance among Chinese Middle School Students: A Cluster-Randomized Controlled Trial

**DOI:** 10.3390/nu9030226

**Published:** 2017-03-02

**Authors:** Xiaoqin Wang, Zhaozhao Hui, Xiaoling Dai, Paul D. Terry, Yue Zhang, Mei Ma, Mingxu Wang, Fu Deng, Wei Gu, Shuangyan Lei, Ling Li, Mingyue Ma, Bin Zhang

**Affiliations:** 1 Department of Public Health, Xi’an Jiaotong University Health Science Center, Xi’an 710061, China; huizhaozhao93@163.com (Z.H.); zymoon95@126.com (Y.Z.); wysun201314195@163.com (M.M.); wangmx601@mail.xjtu.edu.cn (M.W.); 232guwei@mail.xjtu.edu.cn (W.G.); shuangyan724@163.com (S.L.); liling-ch@163.com (L.L.); mamingyue66@163.com (M.M.); zhbin@mail.xjtu.edu.cn (B.Z.); 2 Department of Nursing, Shaanxi Provincial Tumor Hospital, Xi’an 710061, China; Daixling113@126.com; 3 Department of Medicine, University of Tennessee Medical Center, Knoxville, TN 37996, USA; pdterry@utk.edu; 4 Xi’an Tie Yi High School, Xi’an 710000, China; dengfu01@126.com

**Keywords:** fortified milk, micronutrient, middle school students, academic performance, motivated strategies for learning

## Abstract

Many children suffer from nutritional deficiencies that may negatively affect their academic performance. This cluster-randomized controlled trial aimed to test the effects of micronutrient-fortified milk in Chinese students. Participants received either micronutrient-fortified (*n* = 177) or unfortified (*n* = 183) milk for six months. Academic performance, motivation, and learning strategies were estimated by end-of-term tests and the Motivated Strategies for Learning Questionnaire. Blood samples were analyzed for micronutrients. In total, 296 students (82.2%) completed this study. Compared with the control group, students in the intervention group reported higher scores in several academic subjects (*p* < 0.05), including languages, mathematics, ethics, and physical performance at the end of follow-up. Students in the intervention group showed greater self-efficacy and use of cognitive strategies in learning, and reported less test anxiety (*p* < 0.001). Moreover, vitamin B_2_ deficiency (odds ratio (OR) = 0.18, 95% confidence interval (CI): 0.11~0.30) and iron deficiency (OR = 0.34, 95% CI: 0.14~0.81) were less likely in the students of the intervention group, whereas vitamin D, vitamin B_12_, and selenium deficiencies were not significantly different. “Cognitive strategy” had a partial mediating effect on the test scores of English (95% CI: 1.26~3.79) and Chinese (95% CI: 0.53~2.21). Our findings suggest that micronutrient-fortified milk may improve students’ academic performance, motivation, and learning strategies.

## 1. Introduction

More than 2 billion people suffer from micronutrient deficiencies worldwide, including many school-aged children and adolescents in developing countries [1]. A recent systematic review reported that China has a high prevalence of micronutrient deficiencies, and that 55.7%, 45.2%, and 84.7% of children have insufficient iron, vitamin D, and selenium, respectively [2]. For instance, iron deficiency can have a detrimental effect on physical performance in children and adolescents [3]. Vitamin D deficiency in early life may negatively affect neuronal differentiation, axonal connectivity, dopamine ontogeny, and brain structure and function [4]. Retinoic acid, the active metabolite of vitamin A, is tied to processes of neural plasticity, and may influence memory [5,6]. Micronutrient deficiencies have been linked to damaging physical performance [3], impaired cognitive functioning [7], suboptimal learning [8], and poor academic performance [9]. These endpoints, in turn, may lead to an increased risk of adulthood obesity [10,11], living in poverty [12], depression [13], and other psychiatric disorders [14]. Hence, there is a need to identify and evaluate safe, tolerable, and cost-effective nutritional interventions in school children and adolescents.

Food fortification has been an effective public health strategy to decrease micronutrient deficiencies [15,16], but the effect of micronutrient-fortified food on academic performance remains unclear [17,18]. A 2012 literature review [17] identified four studies, none of which showed a positive effect of micronutrient supplementations on school examination grades. On the other hand, a systematic review of randomized controlled trials (RCTs) in 2016 [18] reported a lack of consistency in school performance among students receiving micronutrient interventions. In the latter review, 8 of 19 trials incorporated assessment of academic performance, and one reported significant improvements in mathematics, while no improvement was observed in other academic subjects. Several factors might influence the effect of fortified food on academic performance, such as motivation and learning strategies, which also play important roles in the process of learning and have significant influences on academic performance [19]. In a cross-sectional study, milk intake showed significant positive correlations with testing technique and learning strategy in Korean male high school students [20]. However, there have been few studies investigating the effect of fortified food on both motivated strategies for learning and academic performance.

China has a considerable number of school-aged children and adolescents who would benefit from an integrated nutrition improvement policy approach. In 2011, the General Office of the State Council launched the Nutrition Improvement Program for Rural Compulsory Education Students (NIPRCES), which allots children undergoing compulsory education a daily container of milk and a chicken egg [21]. Although NIPRCES has been implemented for several years, it has not yet been utilized fully in many urban areas, and has yet to be studied for potential effects on school performance. Given this dearth of knowledge, we hypothesized that milk fortified with micronutrients would go further than regular milk in improving micronutrient status, and would positively influence academic performance, motivation, and use of effective studying strategies.

## 2. Materials and Methods

### 2.1. Study Design and Participants

We conducted a cluster-randomized controlled trial among healthy Chinese middle school students, aged 12 to 14 years between June 2015 and January 2016. This study was carried out according to the guidelines laid down in the Declaration of Helsinki and all procedures involving human subjects were approved by the Biomedical Ethics Committee of Xi’an Jiaotong University Health Science Center (Project identification code: 2015-356). Prior to the data collection, written informed consent was obtained from a parent or guardian of all participating students along with verbal assent from each student. The exclusion criteria for children included moderately/severely undernourished children (Body Mass Index (BMI) for age *z*-score < −2 SD) [22], severe anemia (Hemoglobin (Hb) < 8 g/dL), infection (White Blood Cell (WBC) > 10.0 * 10^9^/L), history of food allergies, children consuming nutritional supplements, and those participating in another nutritional program.

**Figure 1:**
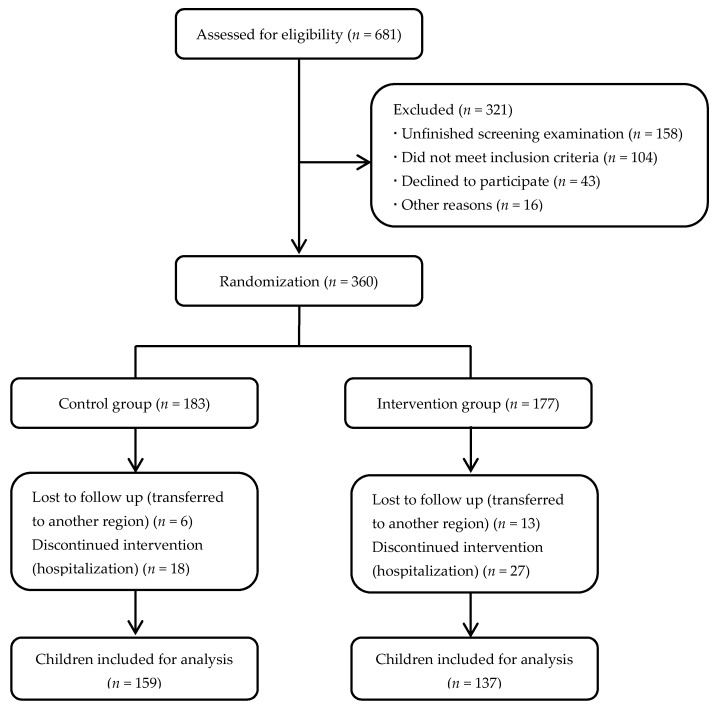
Schematic flow of the participants.

A total of 681 students were recruited from Xi’an Middle School. After excluding 321 students (47.1%) who missed the screening examination, declined to participate, or were deemed ineligible, 360 students were enrolled in the present study. Participating children were allocated to either an intervention group (*n* = 177) or a control group (*n* = 183) with random number table by the research staff, considering each class as a cluster, such that each student in the class, if eligible, would be included. The schematic flow of the participants in the present study is shown in Figure 1. Subjects of the intervention group were given 250 mL micronutrient-fortified milk (Future Star, Mengniu Dairy Company Limited, Hohhot, China) per day for six months; students of the control group were provided pure milk with approximately the same caloric value of the fortified milk (Milk Deluxe, China Mengniu Dairy Company Limited, Hohhot, China) (Table 1). The milk was given to each student by the research assistants, and its consumption was supervised by the students’ teachers. Academic performance, motivation, and learning strategies, and micronutrient status were all assessed at baseline and at the end of follow-up. Children, study investigators, and the data analyst were not blinded to treatment allocation.

### 2.2. Screening Examination

The screening examination included anthropometric measurements and routine blood tests. Body height and weight were measured by trained personnel using standard anthropometric techniques. Subjects removed their shoes, emptied their pockets, and wore indoor clothing. Weight was recorded to the nearest 0.1 kg using a digital weighing scale. Height was measured to the nearest 0.1 cm using a stadiometer from head to foot. The weight and height of each participant were measured twice by study personnel. A third height and/or weight measurement was taken in the rare event that the first two measurements were not in agreement. BMI was derived from weight and height (kg/m^2^), and thereafter BMI *z*-scores were calculated based on growth reference algorithms developed by the World Health Organization (WHO) for children and youth [23].

Hb and WBC counts were also assessed before the intervention to exclude children with severe anemia and infection. Non-fasting venous blood samples were collected in tubes containing anticoagulant (EDTA–K2). Blood samples were stored at 4 °C and analyzed within 4 hours. Hb and WBC counts were measured with an automatic hematology analyzer (XFA6100, PERLONG, Nanjing, China).

### 2.3. Academic Performance

Academic performance was measured using age- and gender-standardized end-of-term test scores retrieved from the school administration system. Academic tests were designed and administered by the Education Bureau of Xi’an, and scores obtained before the intervention were compared with those obtained at the end of follow-up. Test scores were analyzed using a percentage grading system, with 100 as the maximum grade and 60 percent as the minimum passing grade. The subjects of Chinese, mathematics, English, physics, social science, ethics, and physical performance were evaluated in the present study. Physical performance was assessed by a Physical Fitness Test, which includes a 1000-metre race for boys/800-metre race for girls, a 50-metre race, a standing long jump, sit-and-reach exercises, and pull-ups for boys/sit-ups for girls. Performance on the Physical Fitness Test was converted to a percentile score based on the national standard.

### 2.4. Motivation and Learning Strategies

Motivation and learning strategies were assessed by the Motivated Strategies for Learning Questionnaire (MSLQ) [24]. The MSLQ is a 44-item self-reported instrument consisting of three motivational belief subscales (Self-Efficacy, Intrinsic Value, and Test Anxiety), the Cognitive Strategy subscale and the Self-regulation subscale. The Self-Efficacy subscale is constructed by adding the scores of the students’ responses to nine items regarding perceived competence and confidence in performance of class work. The Intrinsic Value subscale consists of nine items concerning intrinsic interest, perceived importance of course work, as well as preference for “challenge” and mastery of goals. Four items concerning worry about, and cognitive interference on, academic tests were used in the Test Anxiety subscale. The Cognitive Strategy Use subscale consists of 13 items pertaining to the use of rehearsal strategies, elaboration strategies, and organizational strategies. A Self-Regulation subscale was constructed from nine metacognitive and effort management items. Scores for each subscale were computed by summing the scores of specific items. Several items within the MSLQ are negatively worded and must be reversed before the respective score is calculated. Prior to the present study, the psychometric properties of the MSLQ were examined by a questionnaire survey with 30 subjects who did not participate in the present study, indicating a sound reliability (Cronbach’s alpha = 0.79).

### 2.5. Micronutrient Status

Non-fasting venous blood specimens were collected by professional phlebotomist. Serum and plasma samples were separated within 4 hours of collection and stored at −80 °C until analysis. Micronutrient status was measured at baseline and at the end of follow-up for serum ferritin (SF), soluble transferrin receptor (sTfR), vitamin D, vitamin B_2_, vitamin B_12_, and selenium. SF, vitamin D, and vitamin B_12_ were measured with electrochemiluminescence technique (Elecsys 2010, Roche Diagnostics, Mannheim, Germany). sTfR was measured using immunoturbidimetry (IMMAGE 800, Beckman Coulter, Carlsbad, America). Vitamin B_2_ was measured by the Erythrocyte Glutathione Reductase Activity Coefficient method using UV-VIS 1800 spectrophotometer by the modified ascorbic acid methodology [25]. The serum selenium levels were determined by atomic fluorescence spectrometry (RGF-8780, Bohui, Beijing, China). All biochemical analyses were carried out at the Micronutrient Laboratory, Division of Nutrition, Xi’an Jiaotong University Health Science Center. SF less than 15 mg/L or sTfR greater than 8.5 mg/L was considered to be a sign of iron deficiency. Deficiencies of vitamin D, vitamin B_2_, vitamin B_12_, and selenium were defined as vitamin D less than 11 ng/mL, the activity coefficient of vitamin B_2_ greater than 1.2 AC, vitamin B_12_ less than 203 pg/mL, and body selenium less than 84.9 mg/mL, respectively. Body iron was calculated as Body Iron (mg/kg) = −[log (R/F ratio) − 2.8229]/0.1207 where R/F ratio = sTfR/SF [26]. 

### 2.6. Statistical Analysis

Data management and data analysis were performed using Epidata (The Epidata Association, Odense, Denmark) and SPSS (Statistical Package for the Social Sciences for Windows, IBM, Armonk, NY, USA) version 23.0. The nominal variables are presented as frequency and proportion. The distribution normality of the quantitative variables was tested by One-Sample Kolmogorov-Smirnov test. The normally distributed variables are presented as mean ± standard deviation (SD). Student’s *t*-tests were performed to analyze the differences in anthropometric parameters such as age, height, weight, and BMI, whereas gender difference and the prevalence of micronutrient deficiencies at baseline between the two groups were tested using Chi-square tests. For categories with small numbers (theoretical frequency < 5), the Fisher’s exact test was used. After the intervention, the prevalence of micronutrient deficiencies was analyzed with logistic regression models. Analysis of covariance (ANCOVA) was used to test differences in academic performance, motivation and learning strategies while adjusting for baseline measures of independent variance. The mediating effects of motivation and learning strategies on academic performance in micronutrients were analyzed with nonparametric Bootstrap methods [27]. Statistical significance was set at *p* < 0.05; all tests were two-sided.

## 3. Results

### 3.1. Demographic and Anthropometric Characteristics of Subjects

The demographic and anthropometric characteristics of the subjects in this study are shown in Table 2. Overall, 137 students in the intervention group (77.4%) and 159 students in the control group (86.9%) completed this study. The mean age of the students at the time of enrollment was 13.2 ± 1.0 years and 13.4 ± 0.9 years in the intervention group and the control group, respectively. The study sample included more girls than boys, although the proportions did not differ significantly by intervention group (*p* = 0.894). There were also no significant differences in age (*p* = 0.071), height (*p* = 0.283), weight (*p* = 0.100), BMI (*p* = 0.252), or BMI *z*-scores (*p* = 0.509) between the two groups.

### 3.2. Micronutrient Deficiencies

Micronutrient deficiencies in students were comparable between the intervention group and the control group at baseline (Table 3). The effects of micronutrient-fortified milk consumption on iron, vitamin D, vitamin B_2_, vitamin B_12_, and selenium deficiencies analyzed with logistic regression models are shown in Table 4. After six months, students in the intervention group were less likely to be iron deficient (odds ratio (OR) = 0.34, 95% confidence interval (CI): 0.14~0.81) and vitamin B_2_ deficient (OR = 0.18, 95% CI: 0.11~0.30) when compared with the control group. However, there was no statistically significant difference in the prevalence of vitamin D, vitamin B_12_, and selenium deficiencies between the two groups.

### 3.3. Academic Performance

The academic scores of trial participants are shown in Table 5. The academic performance of the subjects was comparable between the intervention group and the control group at baseline (*p* > 0.05). Compared with students receiving unfortified milk, students receiving micronutrient-fortified milk showed significantly higher scores in the subjects of Chinese, mathematics, English, ethics, and physical performance (*p* < 0.05), whereas the scores for physics were higher but not statistically significant (*p* = 0.224). No significant difference was observed in social science scores (*p* = 0.428). When modeled as independent variables, both iron and vitamin B_2_ were associated with improved performance in the subjects of Chinese, mathematics, English, ethics, and physical performance *(p* < 0.05).

### 3.4. Motivation and Learning Strategies

Baseline motivation and learning strategy scores were comparable between the intervention groups for self-efficacy, intrinsic value, test anxiety, cognitive strategy, and self-regulation (Table 6). After the intervention, students in the fortified milk group showed higher scores for self-efficacy (*p* < 0.001), and lower scores for test anxiety (*p* < 0.001), than those in the control group. There was no significant difference in scores for “intrinsic value”. Regarding use of learning strategies, students who consumed fortified milk were more likely to incorporate cognitive strategies into their study routines (*p* < 0.001). However, no significant difference was observed in “self-regulation” between the two groups. In addition, the use of cognitive strategies had a partial mediating effect on academic scores in relation to iron and vitamin B_2_, accounting for 29.3% (95% CI: 1.26~3.79) of the improved performance in English and 14.7% (95% CI: 0.53~2.21) for Chinese.

## 4. Discussion

We conducted a cluster-randomized, controlled feeding intervention study to determine the effect of micronutrient-fortified milk versus unfortified milk on academic performance among Chinese middle school students aged 12 to 14 years. The micronutrient-fortified milk intervention raised blood vitamin B_2_ and iron levels, and appeared to increase academic performance, physical performance, learning motivation, and the successful use of study strategies.

Children in our study who consumed micronutrient-fortified milk had significantly higher academic performance than those who consumed unfortified milk, not entirely consistent with findings in previous studies [9,17,28,29]. A recent literature review found that there was a correlation between micronutrients and the academic performance in school children [9]. However, another systematic review concluded no positive effect of multiple micronutrient supplementations on school examination grades [17]. For specific micronutrients, one cross-sectional study showed that iron insufficiency was related to disadvantages in learning, and insufficient serum iron concentration was correlated with significantly lower mathematic scores in female students (r = 0.628) [27]. Another interventional study suggested that improving iron status through fortified rice can enhance school performance (*p* = 0.022) [29]. In addition, a systematic review concluded that serum vitamin B_12_ levels were associated with cognitive function [30], which may further influence academic performance in school children [31]. Moreover, Babur demonstrated the negative effect of selenium deficiency on learning and memory in adult rats [32].

The beneficial effect on academic performance in the present study can be attributed to improved vitamin B_2_ and iron status. Students who consumed fortified milk showed less iron deficiency, although iron was not added to the milk. The reason for this finding is unclear, although vitamin B_2_ may influence iron status, possibly at the level of iron absorption [33]. Micronutrient levels have been linked in Indian school children to improved cognitive and physical performance [34]. Iron may alter the intracellular signaling pathways and electrophysiology of the developing hippocampus, the brain region responsible for recognition, learning, and memory [35]. In addition, we found that students in the intervention group had significantly higher physical performance than what was observed in controls. This may be attributed to improved iron status and oxygen carrying capacity in hemoglobin [36].

Another possible mechanism contributing to the improved academic performance might be the mediating role of learning strategies [37]. The present study found that students in the intervention group were more self-efficacious and had less test anxiety, and were also more likely to use cognitive strategies in the process of learning. Students’ perception of self-efficacy and the evaluation of their own competence were significantly and positively related to academic achievement [38,39]. In a study of Finnish upper secondary school students [40], a statistically significant correlation was found between test anxiety levels and academic performance. Abdollahpour [41] also revealed that using cognitive strategies were positively correlated particularly with math achievement among male high school freshmen. Similarly, Zahrou [42] found that the consumption of fortified milk has a favorable effect on cognitive ability. Our data suggest that use of cognitive strategies may mediate the association between nutrient status and academic performance. Taken together with the results of these previous studies, our findings suggest a potentially long-term benefit to school-aged children from a relatively inexpensive intervention. 

There are two strengths in the present study. Firstly, we not only examined the effect of fortified milk on students’ nutritional status, but also on their academic performance, motivation, and use of learning strategies. Secondly, the cluster-randomized controlled trial design allows control of both measured and unmeasured confounding factors. Furthermore, the cluster-randomized design minimizes the possibility of contamination between the intervention and control group [43], because there is less opportunity to exchange the milk product for the participating students.

Several limitations of our study must be considered. Information on dietary factors other than nutritional supplements during the intervention period was not collected. Therefore, we cannot be certain that our results were not influenced by unmeasured dietary factors. Similarly, we did not account for factors such as “self-concept” [44], physical fitness [45], and cell phone use [46], which have been found to affect academic achievement. Lastly, the six-month follow-up period precluded the examination of longer-term effects of micronutrient-fortified milk on academic outcomes.

## 5. Conclusions

In conclusion, the results of the present study suggest that the consumption of micronutrient-fortified milk may improve academic performance, motivation, and learning strategies in Chinese school children. If our results are confirmed in future studies, additional studies will be needed to elucidate the underlying mechanisms and to identify subgroups of undernourished student populations that are most likely to benefit from this intervention.

## Figures and Tables

**Table 1:** Nutrient composition of the micronutrient fortified milk and pure milk in the present study.

Nutrients	Units	Fortified Milk per 100 mL	Pure Milk per 100 mL	FAO/WHO RNI for 10–18 Years ^a^	Chinese DRIs EER/RNI/AI/EAR for 11–14 Years ^b^
Energy	KJ	332	309	—	7530/8580 ^c^
Protein	g	3.1	3.6	—	55/60
Fat	g	3.6	4.4	—	<60
Carbohydrate	g	8.6	5.0	—	150
Sodium	mg	58	65	—	1400
Vitamin A	μg RE	78	0	600	630/670 ^c^
Vitamin D	μg	1.5	0	5	10
Vitamin E	mg α-TE	2.0	0	7.5/10.0 ^c^	13
Vitamin B_2_	mg	0.09	0	1.0/1.3 ^c^	1.1/1.3 ^c^
Pantothenic acid	mg	0.2	0	5.0	4.5
Phosphorus	mg	70	0	—	640
Calcium	mg	100	120	1300	1200
Zinc	mg	0.34	0	7.2/8.6 ^c^	9.0/10.0 ^c^

RNI: Recommended Nutrient Intake; DRIs: Dietary Reference Intakes; EER: Estimated Energy Requirement; AI: Adequate Intake; EAR: Estimated Average Requirement; RE: Retinol Equivalent; α-TE: α-Tocopherol Equivalent; ^a^ Food and Agriculture Organization of the United Nations (FAO) and World Health Organization (WHO), 2004. Vitamins and Mineral Requirements in Human Nutrition. Second Edition; ^b^ Chinese Nutrition Society, 2013. Chinese Dietary Reference Intakes; ^c^ Female/Male.

**Table 2:** Demographic and anthropometric characteristics of subjects in the intervention and control groups.

Variables	Intervention (*n* = 137)	Control (*n* = 159)	*t/X*^2^	*p*
Age (years)	13.2 ± 1.0	13.4 ± 0.9	1.811	0.071
Gender			0.018	0.894
Male (*n* (%))	38 (27.7)	43 (27.0)		
Female (*n* (%))	99 (72.3)	116 (73.0)		
Height (cm)	163.9 ± 1.7	163.7 ± 1.5	1.075	0.283
Weight (kg)	58.8 ± 4.1	58.1 ± 3.2	1.648	0.100
BMI (kg/m^2^)	21.2 ± 0.8	21.1 ± 0.7	1.147	0.252
BMI *z*-scores	0.1 ± 1.3	0.2 ± 1.3	0.660	0.509

BMI: Body Mass Index.

**Table 3:** Prevalence of iron, vitamin D, vitamin B_2_, vitamin B_12_, and selenium deficiencies in subjects at baseline between the intervention and control group.

Micronutrients	Intervention *(n* = 137)	Control (*n* = 159)	*X*^2^	*p*
Iron deficiency	10 (7.3)	13 (8.3)	0.079	0.779
Vitamin D deficiency	5 (3.6)	5 (3.1)		0.999 *
Vitamin B_2_ deficiency	127 (92.7)	145 (91.2)	0.224	0.636
Vitamin B_12_ deficiency	12 (8.8)	15 (9.4)	0.040	0.841
Selenium deficiency	68 (49.6)	77 (48.4)	0.043	0.836

* *p* value was compared using Fisher’s exact test.

**Table 4:** The effects of micronutrient-fortified milk on the prevalence of iron, vitamin D, vitamin B_2_, vitamin B_12_, and selenium deficiencies.

Micronutrients	Intervention (*n* = 137)	Control (*n* = 159)	Adjusted OR	95% CI	*p*
Iron deficiency	7 (5.1)	22 (13.8)	0.34 ^a^	0.14~0.81	0.012
Vitamin D deficiency	6 (4.4)	8 (5.0)	0.87 ^b^	0.29~2.56	0.792
Vitamin B_2_ deficiency	31 (22.6)	99 (62.3)	0.18 ^c^	0.11~0.30	0.000
Vitamin B_12_ deficiency	4 (2.9)	3 (1.9)	1.56 ^d^	0.34~7.11	0.708
Selenium deficiency	53 (38.7)	59 (37.1)	1.07 ^e^	0.67~1.71	0.780

OR: Odds Ratio; CI: Confidence Interval; ^a^ Adjusted by gender, age, BMI, vitamin D, vitamin B_2_, vitamin B_12_, selenium; ^b^ Adjusted by gender, age, BMI, iron, vitamin B_2_, vitamin B_12_, selenium; ^c^ Adjusted by gender, age, BMI, iron, vitamin D, vitamin B_12_, selenium; ^d^ Adjusted by gender, age, BMI, iron, vitamin D, vitamin B_2_, selenium; ^e^ Adjusted by gender, age, BMI, iron, vitamin D, vitamin B_2_, vitamin B_12_.

**Table 5:** Academic scores of the end-of-term tests between the control and intervention group in middle school students.

Subjects	Intervention (*n* = 137)		Control (*n* = 159)		F	*p*	F’	*p*’
	Baseline	Post-Trial	Baseline	Post-Trial				
Chinese	72.1 ± 2.0	81.2 ± 2.2	72.3 ± 2.1	78.5 ± 2.0	127.852	0.000	127.395	0.000
Mathematics	82.8 ± 2.0	86.1 ± 2.1	82.4 ± 2.0	85.6 ± 2.0	9.416	0.002	8.013	0.005
English	73.0 ± 2.0	84.1 ± 1.9	72.6 ± 2.0	79.3 ± 2.0	497.398	0.000	483.216	0.000
Physics	62.6 ± 2.1	70.0 ± 2.0	62.2 ± 2.2	69.5 ± 2.4	1.766	0.185	1.484	0.224
Social science	81.3 ± 2.1	84.9 ± 2.0	80.9 ± 1.8	85.2 ± 2.2	0.591	0.443	0.629	0.428
Ethics	72.6 ± 1.9	77.8 ± 2.1	72.4 ± 2.1	74.9 ± 2.0	127.497	0.000	127.637	0.000
Physical performance	68.7 ± 3.7	83.3 ± 4.7	69.3 ± 3.4	78.5 ± 4.4	79.162	0.000	59.090	0.000

F’: Adjusted by gender, age, BMI, iron, vitamin D, vitamin B_2_, vitamin B_12_, selenium, self-efficacy, intrinsic value, test anxiety, cognitive strategy, and self-regulation. The effect sizes (Eta Square) are 0.303, 0.027, 0.623, 0.005, 0.002, 0.004, and 0.168 for Chinese, mathematics, English, physics, social science, ethics, and physical performance, respectively.

**Table 6:** Motivation and learning strategy scores between the control and intervention group in middle school students.

Dimensions	Score				F	*p*	F’	*p’*
	Intervention (*n* = 137)		Control (*n* = 159)					
	Baseline	Post-Trial	Baseline	Post-Trial				
Self-efficacy	50.3 ± 2.7	52.5 ± 0.9	49.9 ± 2.0	51.9 ± 0.7	19.497	0.000	17.621	0.000
Intrinsic value	50.8 ± 3.3	53.0 ± 2.9	51.3 ± 2.5	52.6 ± 2.7	0.375	0.541	0.285	0.594
Test anxiety	22.0 ± 5.5	20.1 ± 4.3	21.4 ± 4.3	22.8 ± 3.7	41.278	0.000	40.905	0.000
Cognitive strategy	58.2 ± 2.8	61.1 ± 3.1	57.7 ± 2.6	60.1 ± 2.7	15.885	0.000	15.730	0.000
Self-regulation	47.6 ± 3.2	47.5 ± 1.7	48.0 ± 2.5	47.6 ± 2.1	0.987	0.321	1.174	0.279

F’: Adjusted by gender, age, BMI, iron, vitamin D, vitamin B_2_, vitamin B_12_, and selenium. The effect sizes (Eta Square) are 0.057, 0.001, 0.123, 0.051, and 0.004 for self-efficacy, intrinsic value, test anxiety, cognitive strategy, and self-regulation, respectively.

## Abbreviations

The following abbreviations are used in this manuscript:

MSLQ	Motivated Strategies for Learning Questionnaire
EAR	Estimated Average Requirement
NIPRCES	Nutrition Improvement Program for Rural Compulsory Education Students
BMI	Body Mass Index
Hb	Hemoglobin
WBC	White Blood Cell
RNI	Recommended Nutrient Intake
DRIs	Chinese Dietary Reference Intakes
EER	Estimated Energy Requirement
AI	Adequate Intake
SF	Serum Ferritin
STfR	Soluble Transferrin Receptor
SPSS	Statistical Package for the Social Sciences
SD	Standard Deviation
